# I Can’t Hear You Both at the Same Time: A Temporal Dilemma During Inter-Regional Communication

**DOI:** 10.1523/ENEURO.0012-26.2026

**Published:** 2026-08-04

**Authors:** Salil Bhat, Sanne Ten Oever

**Affiliations:** Department of Cognitive Neuroscience, Faculty of Psychology and Neuroscience, Maastricht University, Maastricht 6229EV, The Netherlands

**Keywords:** inter-area communication, neural oscillations, optimal coupling, synchronization

## Abstract

Cognitive functioning depends on the brain’s ability to process sensory information and simultaneously integrate contextual feedback from higher-order regions like the prefrontal cortex (PFC). This requires the sensory cortex (SC) to handle both processes simultaneously. Some phase-coupled oscillator models propose that the scaffolding of neuronal communication occurs via oscillatory coupling of low-frequency oscillations. However, it is often neglected that processing this bidirectional input poses serious temporal constraints on the system. Specifically, is it possible for SC to be coupled to PFC, while at the same time being coupled to the sensory input? In this article, we describe the temporal constraints required to simultaneously process feedforward and feedback information through oscillatory coupling. We adopt a dynamical systems perspective to suggest mechanisms by which phase-coupled oscillator models can achieve optimal temporal dynamics for neural communication while accounting for these temporal constraints. Although initially counterintuitive, our proposed framework indicates that any viable solution of bidirectional phase-based coupling inherently relies on the feedforward scaffolding of neuronal communication. The mechanisms proposed here may generalize to other situations in which brain areas need to cope with bidirectional feedforward and feedback interactions while maintaining phase coupling.

## Significance Statement

Inter-regional phase-based brain communication is subjected to several temporal constraints such as phase excitability, transmission lags and environmental coupling. These constraints raise an interesting temporal dilemma: How can one brain region simultaneously couple with another brain region and the environmental input? In this work, we analyze this problem through the lens of dynamical systems theory. We first lay out the temporal constraints and show that optimal processing requires higher-level areas to anticipate activity in low-level areas. We propose various implementations of anticipation using phase-coupled oscillators and validate our solutions with numerical simulations. Investigating temporal dynamics of inter-area coupling is essential for understanding how the brain transfers information across neural regions while processing continuous input from the environment.

## The Coordination Paradox

**Figure 1.**
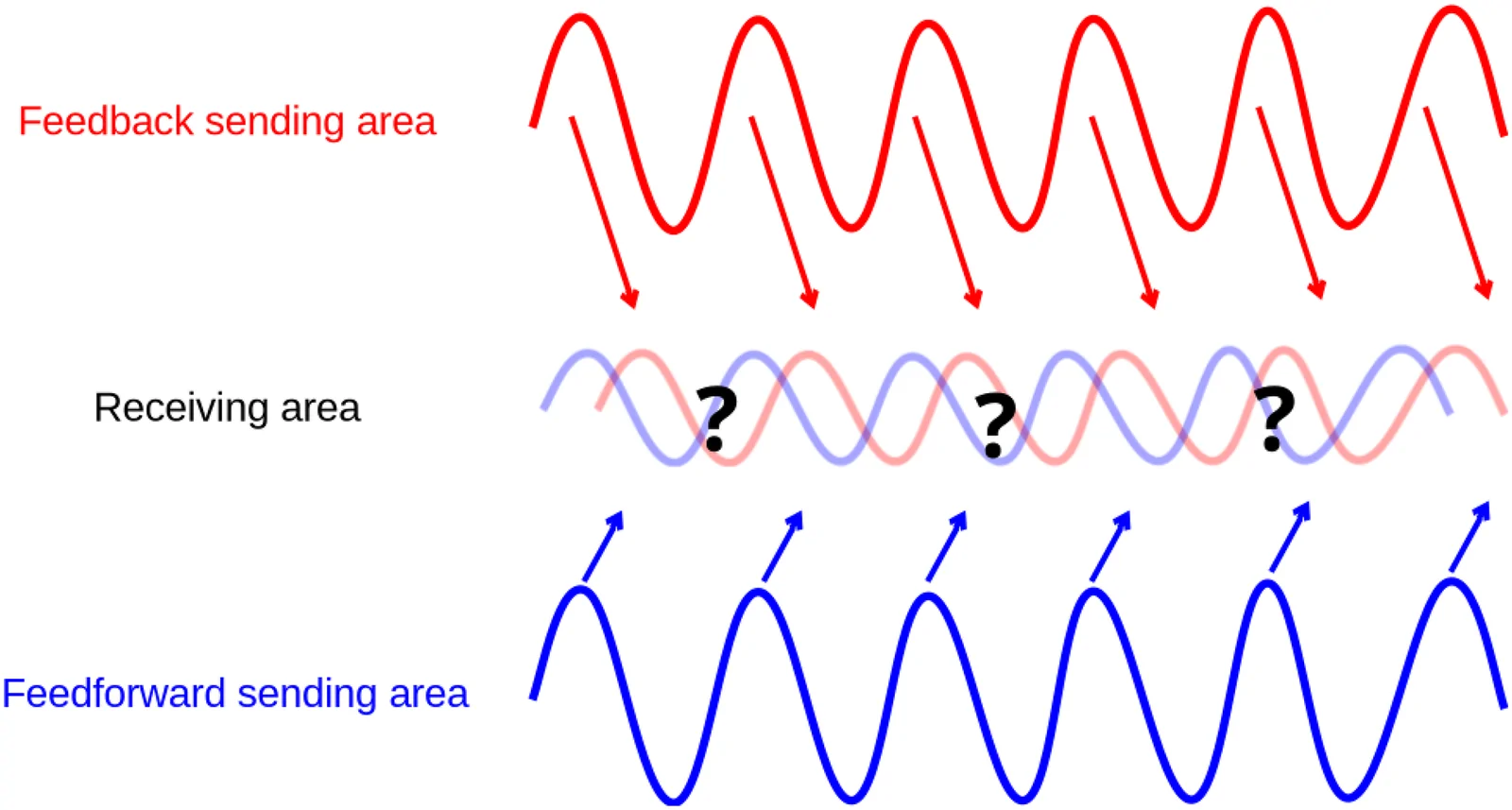
How can a receiving area simultaneously process feedforward and feedback information?.

Neural oscillations are ubiquitous to the brain and are one of the central elements of the brain’s natural temporal dynamics. These oscillations reflect population-level activity of neurons rhythmically de- and hyper-polarizing their membrane potentials, often leading to action potentials being clustered at de-polarized phases of an oscillatory cycle (Buzsaki and Draguhn, 2004; Lakatos et al., 2007; Haegens et al., 2011). The observation of phase-clustered neural organization has led to the proposal that neural oscillations provide a mechanism for inter-areal communication in the brain (Fries, 2005; Schnitzler and Gross, 2005; Siegel et al., 2012; Cariani and Baker, 2022; Trajkovic et al., 2025), with theta band activity often implicated in long-range communication (Fiebelkorn et al., 2018). Phase coupling refers to the idea that optimal processing of incoming information is achieved by aligning phases of different brain areas that are relevant for a given task. This mechanism should work for processing bottom-up sensory input (Schroeder and Lakatos, 2009b; Haegens and Golumbic, 2018; Keitel et al., 2025), but also for processing feedback arriving from higher-order cortical areas (Alamia and VanRullen, 2019; Pang et al., 2020). It is still unclear whether this mechanism works in situations when a single brain area must simultaneously process bottom-up and top-down information at the same time; for example, information about incoming sensory input as well as higher-order feedback information. To illustrate, the sensory cortex (SC) receives feedforward information from subcortical structures that process sensory input, and at the same time, it receives feedback information from the prefrontal cortex (PFC). During most natural situations with continuous sensory input, SC faces a paradoxical situation where it needs to send sensory information to PFC while receiving feedback at the same time from PFC (Fig. 1). This simultaneous feedforward–feedback processing is a situation faced not only in SC but also in many other regions. In this article, we shed light on this paradoxical situation and propose possible dynamical systems solutions to how the brain might be dealing with this temporal paradox. The aim of the current article is to find a solution in which information is integrated not only at the same time but also at the same anatomical location (e.g., neurons or neuronal population), which is in line with findings showing that feedback directly influences how neuronal populations process bottom-up sensory input (Larkum et al., 2018; Mikulasch et al., 2023; also see Box 1). We limit ourselves to solutions that fit within the framework of the phase-coupled oscillators, leaving open possible alternative solutions to optimized communication (Jacobs et al., 2007; Lee et al., 2013; Larkum et al., 2018; Schneider et al., 2021; Mikulasch et al., 2023).

**Box 1**: Feedback through segregated frequency or anatomical channels?

The goal of the current manuscript is to find a mechanism by which bidirectional information flow can converge both spatially and temporally at the same anatomical location. This assumption of anatomical precision is motivated by evidence from early sensory processing, where incoming sensory signals are shaped by contextual information at the earliest input stages (Zipser et al., 1996; Petro et al., 2017). If context modulates sensory processing at these stages, then feedforward and feedback signals must ultimately converge in anatomical locations where sensory input is first processed. Such direct spatiotemporal convergence is not readily accounted for in many existing models.

Some proposals assume a layer-specific separation of feedforward and feedback processing (Felleman and Van Essen, 1991; Markov et al., 2014). While such separation is supported anatomically, it leaves unresolved where and how contextual feedback influences input-layer processing. Other models suggest that feedforward and feedback signals are segregated by frequency bands—for example, gamma for feedforward signaling and alpha/beta for feedback (Bastos et al., 2015a; Michalareas et al., 2016). However, this scheme precludes precise temporal convergence, as signals carried in different frequency bands cannot achieve sustained phase alignment. While different frequencies can have an overall consistent relationship through nesting, transmission delays will still misalign the feedforward and feedback input with each other. Alignment at high excitable phases will therefore only be achieved in one direction and not both directions at the same time. Moreover, the role of theta oscillations in these proposals is unclear. A further proposal posits that feedforward and feedback inputs are anatomically segregated within single neurons, with basal dendrites receiving feedforward input and apical tufts receiving feedback (Larkum et al., 2018; Mikulasch et al., 2023). However, both feedforward and feedback inputs remain subject to transmission delays and may not integrate at the same optimal phase. Thus, the dendritic segregation facilitates spatial feedforward and feedback inputs, but it does not guarantee their temporally aligned integration at optimal phases.

In sum, while modeling large-scale brain dynamics as phase-coupled oscillators may be an oversimplification, a fundamental challenge remains: to identify a mechanism that allows feedforward and feedback information to converge in both space and time despite transmission delays. Any model for feedforward/feedback processing must be explicit about when and where neural processing deviates from optimal oscillatory regimes, that is, when information is not integrated at the most excitable phases of ongoing activity.

## Theta Oscillations: The Brain’s Long-Distance Relationship

For most behavioral tasks, the brain uses a widespread network of connected regions across cortical and subcortical structures (Liu et al., 2022). It is still unclear how the brain maintains the coupling between regions that are a part of a task-specific network. One prominent proposal is that regions couple their phases to each other (Fries, 2005), a phenomenon that has been shown for several frequency bands (Bastos et al., 2015b; Michalareas et al., 2016). Specifically for long-range connections, it seems that slower frequencies around theta (4–8 Hz) are involved (Von Stein and Sarnthein, 2000; Mizuhara and Yamaguchi, 2007; Cavanagh and Frank, 2014; Ekstrom and Watrous, 2014; Von Lautz et al., 2017; Fiebelkorn et al., 2018). Electrode arrays covering distant cortical sites make it possible to show phase coupling. Using these arrays, theta oscillations are shown to connect across sensory and frontal sites (Kawasaki et al., 2014; Zhang et al., 2018; Mohan et al., 2024). Low-frequency oscillations are proposed to provide a temporal scaffold that organizes local information processing and enables information transfer across distributed brain regions (Zhang et al., 2018; Fiebelkorn and Kastner, 2019; Trajkovic et al., 2025). According to this framework, information is encoded during phases of heightened excitability of the theta rhythm and is subsequently communicated across cortical areas. This information is likely represented in higher-frequency activity, such as beta (15–25 Hz) and gamma (30–200 Hz) bands, consistent with the well-documented coupling between the phase of low-frequency oscillations and the power of higher-frequency activity (Von Stein and Sarnthein, 2000; Lakatos et al., 2005; Canolty et al., 2006; Schroeder and Lakatos, 2009a) as well as the greater spatial specificity generally attributed to higher-frequency activity (Zheng and Colgin, 2015; Von Lautz et al., 2017; Cariani and Baker, 2022). In the current manuscript, we focus on the temporal scaffold rather than the information content, and therefore we will not focus on these higher frequencies (but see Box 2).

**Box 2**: Where is the feedback?

Feedback from PFC is crucial for providing contextual information. The question we raise in this article is: what is the optimal moment in time when feedback can happen such that processing is still optimized given the temporal constraints? We use theta oscillations as a temporal reference frame, aligning with evidence showing long-range communication in the theta band (Fiebelkorn et al., 2018). It might be counterintuitive that we find feedforward coupling schemes; however, we do not claim that feedback does not take place. Long-range, large-scale couplings in theta are unlikely to encode content-specific feedback information due to their coarse spatial specificity. Instead, they form a perfect scaffold for bidirectional communication flow (Lakatos et al., 2005; Schroeder and Lakatos, 2009b; Fiebelkorn and Kastner, 2019). Considering that higher frequencies are modulated by these slow dynamics (Lakatos et al., 2005; Canolty et al., 2006; Schroeder and Lakatos, 2009a), it is likely that the information content of the feedback is present in the higher frequencies (Zheng and Colgin, 2015; Von Lautz et al., 2017; Cariani and Baker, 2022). Thus, while information exchange itself can be bidirectional, the slow oscillatory dynamics may primarily define a unidirectional temporal reference frame (scaffold) within which this exchange takes place.

Importantly, the relative phase of low-frequency oscillations in SC and PFC is not necessarily the same, and SC has been reported to lead or lag PFC depending on the task at hand (Roland et al., 2006; Muller et al., 2018; Zhang et al., 2018; Mohan et al., 2024). This is not surprising, considering transmission delays that exist across different neural regions. Phase leads and lags of SC relative to PFC have been implied to relate to feedforward or feedback processing, respectively (Van Kerkoerle et al., 2014; Trajkovic et al., 2025). Note, however, that from a dynamical systems point of view a lag or lead does not necessarily imply directionality (also see Section “Feedback from the Future?”), highlighting the need for more modeling and rigorous causal analyzes.

## So, What’s the Catch?

The catch is that most findings on low-frequency oscillations disassociate feedforward and feedback as separate windows of information flow (Alamia and VanRullen, 2019; Pang et al., 2020). This would suggest that SC should operate in two distinct regimes: feedforward and feedback processing. However, in most cases, SC does not operate in separate regimes but needs to process sensory input and contextual feedback at the same time. Khorsand et al. (2015) argue that, mechanistically, feedforward and feedback processes cannot be separated as different processing stages as they occur at the same time and influence the neural activity of the same regions through overlapping mechanisms. It has also been shown that although feedback and feedforward processes are characterized by distinct activity patterns, they coexist with shifting dominance and not as exclusive or separate regimes (Semedo et al., 2022).

This creates an interesting dilemma within the framework of phase coupling oscillators: How can SC optimally receive contextual feedback from PFC without decoupling from the sensory stimulus? Phase-coupled frameworks propose that SC couples with sensory input and forwards this information to higher-order prefrontal areas. However, at the same time, SC must also couple to prefrontal areas to receive contextual information. To do so, it needs to closely follow PFC activity, which may lead to a decoupling from the sensory input. When following common implementations from oscillatory coupling models, it is not trivial how SC can be coupled to PFC while at the same time being coupled to the sensory input. To phrase it differently, both the environment and PFC are allowed to speak to SC at the same time, but SC might struggle a lot to understand them simultaneously.

In this article, we outline the temporal constraints grounded in the literature that are required to satisfy optimal inter-area communication via phase alignment. We highlight how current phase-coupled models of simultaneous feedforward–feedback communication do not satisfy the temporal constraints, based on a dynamical systems perspective using computational models of coupled oscillators. Subsequently, we provide possible mechanistic solutions, based on previous studies and dynamical systems theories, that could potentially satisfy the temporal constraints. The temporal constraints and possible mechanistic solutions are based on the assumption of communication through phase coupling, rather than biophysical information transfer. Additionally, in our constraints we assume that the same information has to arrive at the same time at the same anatomical location. All the computational models, visualizations of their dynamics, and their mathematical descriptions are available at: https://github.com/tedyUM/CommunicationDilemma_models. They are intended as a conceptual description of the problem and possible solutions rather than a fully specified computational model with strong empirical validation. The article is designed to be accessible to a broad audience without requiring a detailed understanding of the underlying mathematics.

## Constraint #1 : I Cannot Talk All the Time

**Figure 2.**
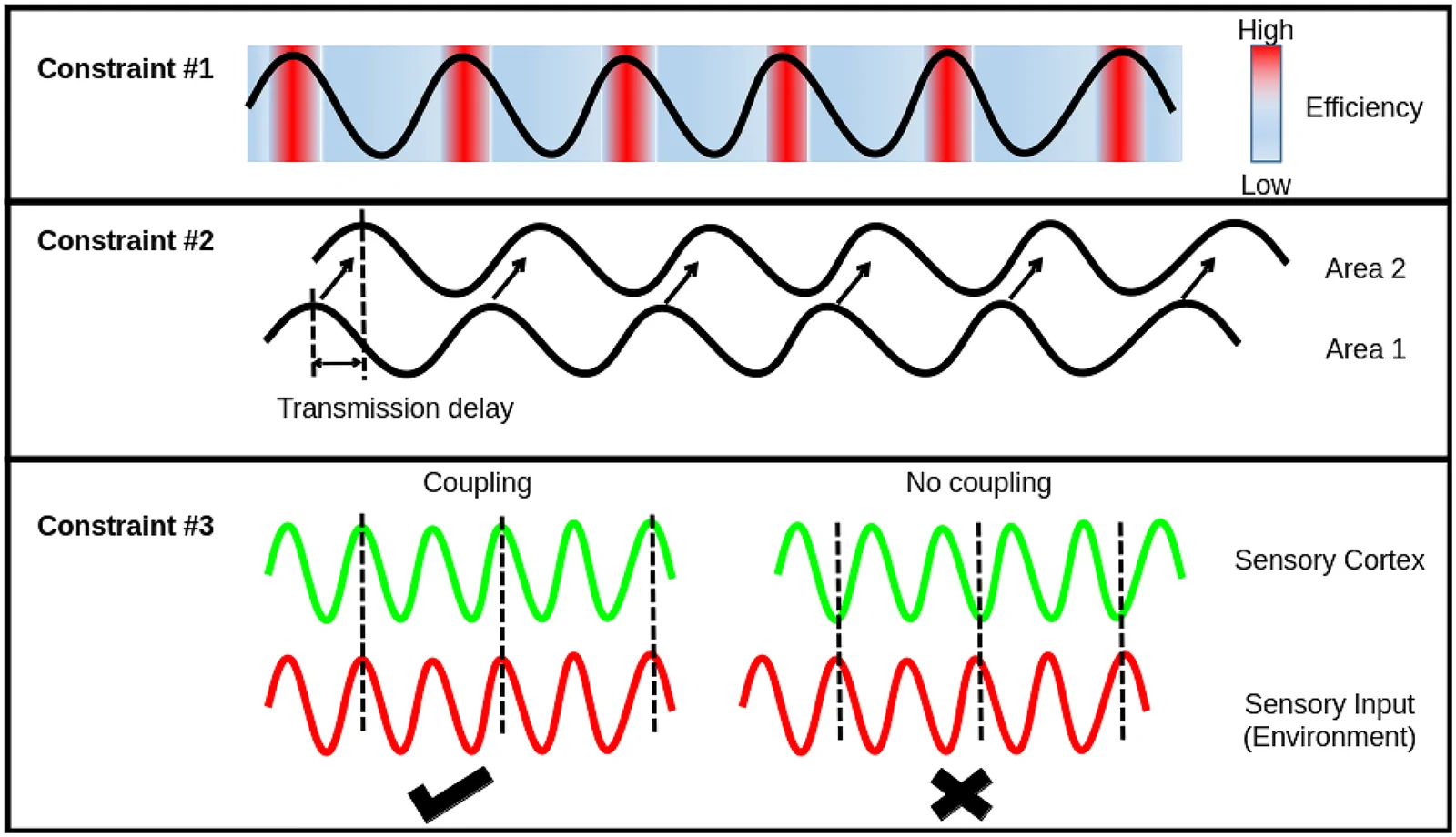
Temporal constraints governing optimal inter-area brain communication.

The observation that oscillations modulate membrane potentials of neuronal populations has led to the suggestion that input arriving at higher excitable phases are amplified, whereas input arriving at less excitable phases are suppressed (Schroeder et al., 2008; Fig. 2). Following this logic, regions communicating with each other should align their excitable phases such that information arrives at the most excitable phase of a receiving group (Fries, 2005; Singer, 2009; Park et al., 2015). This has been proposed most prominently for gamma with the communication through coherence theory (Fries, 2005), but its implications are also relevant for the neuronal scaffolding by low-frequency oscillations (Siapas et al., 2005; Lakatos et al., 2007; Schroeder and Lakatos, 2009b; Fiebelkorn and Kastner, 2019). For example, Lakatos et al. (2007) show that somatosensory input modulates the phase of ongoing theta oscillations such that auditory inputs arrive at the highly excitable phase. Note, however, that alongside positive findings, several studies report null effects for proactive sensory alignment and behavioral oscillatory gating (Haegens and Golumbic, 2018; Keitel et al., 2022), and there are well-founded concerns about interpreting coherence measures indiscriminately as markers of oscillatory alignment (Schneider et al., 2021). Nonetheless, there is still a plethora of studies showing that the phase at which information arrives has influence on neuronal processing (Lakatos et al., 2007; Dugué et al., 2011; Haegens et al., 2011; Zoefel et al., 2018; Zhang and Frohlich, 2022; Ten Oever et al., 2024; Keitel et al., 2025), suggesting that the brain does respond differently depending on the oscillatory state at which information arrives (Schoffelen et al., 2005; Schroeder et al., 2008; Singer, 2009; Deco and Kringelbach, 2016; Mohapatra et al., 2025).

## Constraint #2: We Are Expecting Some Delays

Inter-area communication takes time, and the transfer of information from one area to another is subjected to transmission delays. While polysynaptic connections between distant areas of the brain require up to 50 ms (Valera et al., 1981), the delays reported from low-frequency propagation can be of the order of hundreds of milliseconds (Muller et al., 2018; Zhang et al., 2018). A sending area needs to account for these delays such that information arrives at the receiving area’s excitable phase following constraint #1 (Fries, 2005).

## Constraint #3: I Cannot Zone Out from the Environment

If SC needs to process incoming input, it cannot unreservedly decouple from sensory input, as it would reduce processing efficiency of the external input. Additionally, the coupling between SC and sensory input is necessarily unidirectional from the sensory input to SC as the sensory input represents the changes happening in the external world. Efficient feedforward processing of incoming information therefore requires continuous tracking of the temporal dynamics of the environment through neural coupling (Lakatos et al., 2008; Ten Oever et al., 2017; Keitel et al., 2018). Note that we represent the sensory input as a sinusoidal signal for simplicity. It should be interpreted as a conceptual representation of a temporally structured input rather than a mechanistic description of environmental dynamics.

## Catch Me If You Can

**Figure 3.**
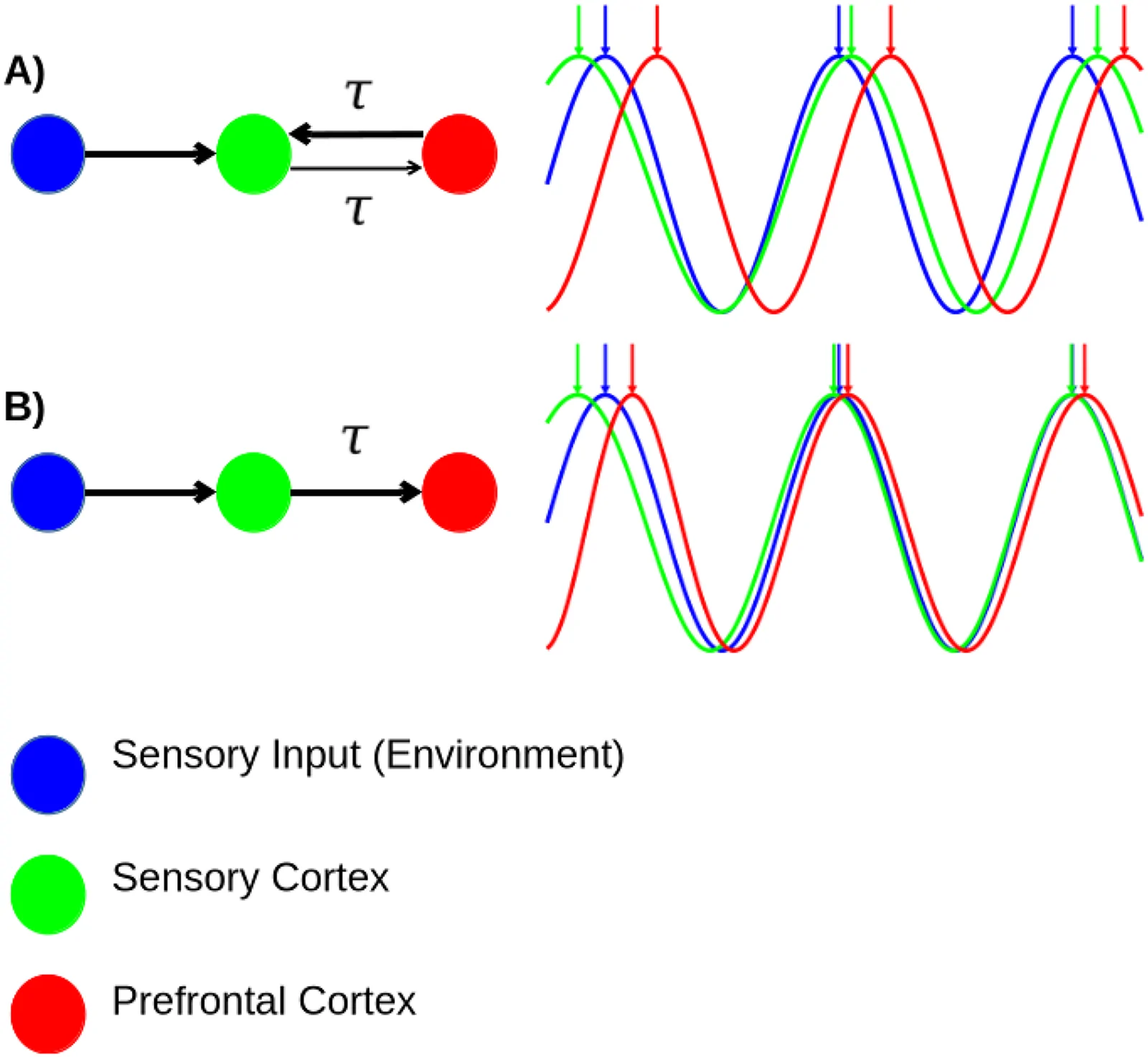
Problems with feedforward or feedback mechanisms. The plots are sinusoids of phases of simulated coupled oscillators. *τ* is the transmission delay. Initial phase conditions are random and the convergent behaviors can be observed at the last cycle. ***A***, Simultaneous feedforward–feedback coupling with transmission delay. In order to strongly couple with PFC and sensory input (thicker arrow indicating stronger coupling) , sensory cortex (SC) decouples with both. A strong coupling to the SC would make the PFC fall behind and information would not arrive to SC on the most excitable point (constraint #1). A strong coupling with PFC would make SC decouple with the sensory input (constraint #3). ***B***, Purely feedforward scheme with transmission delay. In this case, SC couples with sensory input, but the PFC lags behind SC, which would prevent feedback arriving at the most excitable phase (constraint #1).

The most prevalent view of inter-area communication through phase coupling is based on simultaneous feedforward and feedback communication (Ekstrom and Watrous, 2014; Bastos et al., 2015b; Michalareas et al., 2016; Fiebelkorn and Kastner, 2019; Schneider et al., 2021). However, a simultaneous feedforward–feedback coupling would pose a serious threat to the temporal constraints. To illustrate this conceptually, we model each brain area with a Stuart–Landau oscillator, also known as a Hopf model in neuroscience (Ponce-Alvarez and Deco, 2024). It is important to note that the specific choice of the oscillators is made only to describe the dynamics phenomenologically, rather than explaining bio-physically realistic dynamics. Since we are interested in the timing of the dynamics, we reduce the amplitude coupled Stuart–Landau oscillator to phase-coupled oscillator. (Please refer to github link for more details. Note that the logic of the models can be understood in this manuscript without understanding the models.) Since we are only interested in inter-area communication, for simplicity, we ignore that lag between the sensory input and SC. This also makes the visualization easier to interpret and has no consequences on the coupling schemes demonstrated here. In Figure 3*A* we visualize a feedforward–feedback scheme with a relatively strong weight on the feedback. In this visualization, SC should phase align perfectly with the input (as there is no lag between the input and the SC). However, the SC lags the input suggesting a decoupling from the sensory input, therefore not fulfilling constraint #3 (continuous input tracking). This is necessarily true as SC tries to follow PFC (due to the feedback coupling) at the cost of following the sensory input. The situation is similar to a tug-of-war, where SC is pulled both ways and the relative strength of the feedforward and feedback coupling determines the winner. Based on this, it seems that no strong feedback connection can be in place to maintain the neuronal scaffolding as the feedback pulls the SC out of sync with the sensory input. However, a purely feedforward scheme will also not work (Fig. 3*B*). Due to transmission delays, PFC phase lags SC. If PFC in this scheme would send feedback (with again transmission delays), the information will not arrive at the highly excitable phase of SC. This scheme fulfills constraint #3 (continuous input tracking), but not constraint #1 (phase alignment) and #2 (transmission lags) as it will be too late for PFC to provide any contextual feedback. An optimal coupling scheme should allow sensory cortex to couple strongly with sensory input, while at the same time allowing PFC to provide contextual feedback to SC at the right phase.

## Feedback from the Future?

As shown in the previous section, optimal phase-coupled communication cannot be achieved under a simultaneous feedforward–feedback scheme. It has been reported that different brain areas synchronize with time lags (Salazar et al., 2012), which could be a result of these transmission delays (Bastos et al., 2015b). However, there are also reports of different brain areas synchronizing with (near) zero time lag (Roelfsema et al., 1997; Gollo et al., 2011; Osorio and Assaneo, 2025). It still remains a mystery how oscillating neural systems can couple under transmission delays and with unexplainable (near-zero) time lags found in the data. From a dynamical systems perspective, because of these time lags, the feedback would arrive at the wrong time. On top of that, coupling with this feedback information would break constraint #3 (continuous input tracking). And hence SC needs to be connected to PFC in a feedforward motif. However, because of time lags, a simple feedforward coupling would fail to orchestrate optimal communication (Fig. 3*B*). One possible solution to this temporal paradox could be that through some feedforward mechanism, PFC acts before SC, such that it has enough time to provide contextual feedback at the right moment (highly excitable phase). This would then also explain (near) zero-lag synchronization. We propose here, multiple feedforward motifs of dynamical systems that would allow PFC to act ahead of SC.

## Motif #1: Anticipation Through Inhibitory Loops

One way in which PFC can act before SC without dictating its activity is if PFC actively anticipates the activity in SC. Various studies show that frontal theta exhibits some form of anticipatory behavior (van Driel et al., 2015; Zamorano et al., 2020; Martínez-Molina et al., 2024; Jung et al., 2025). For example, Osorio and Assaneo (2025) report negative lags in the inferior frontal gyrus in the slow frequency band (1–8 Hz), during passive movie watching, which could be an indication of anticipation. However, any formal coupling dynamics of this anticipation have not been proposed, let alone empirically tested.

**Figure 4.**
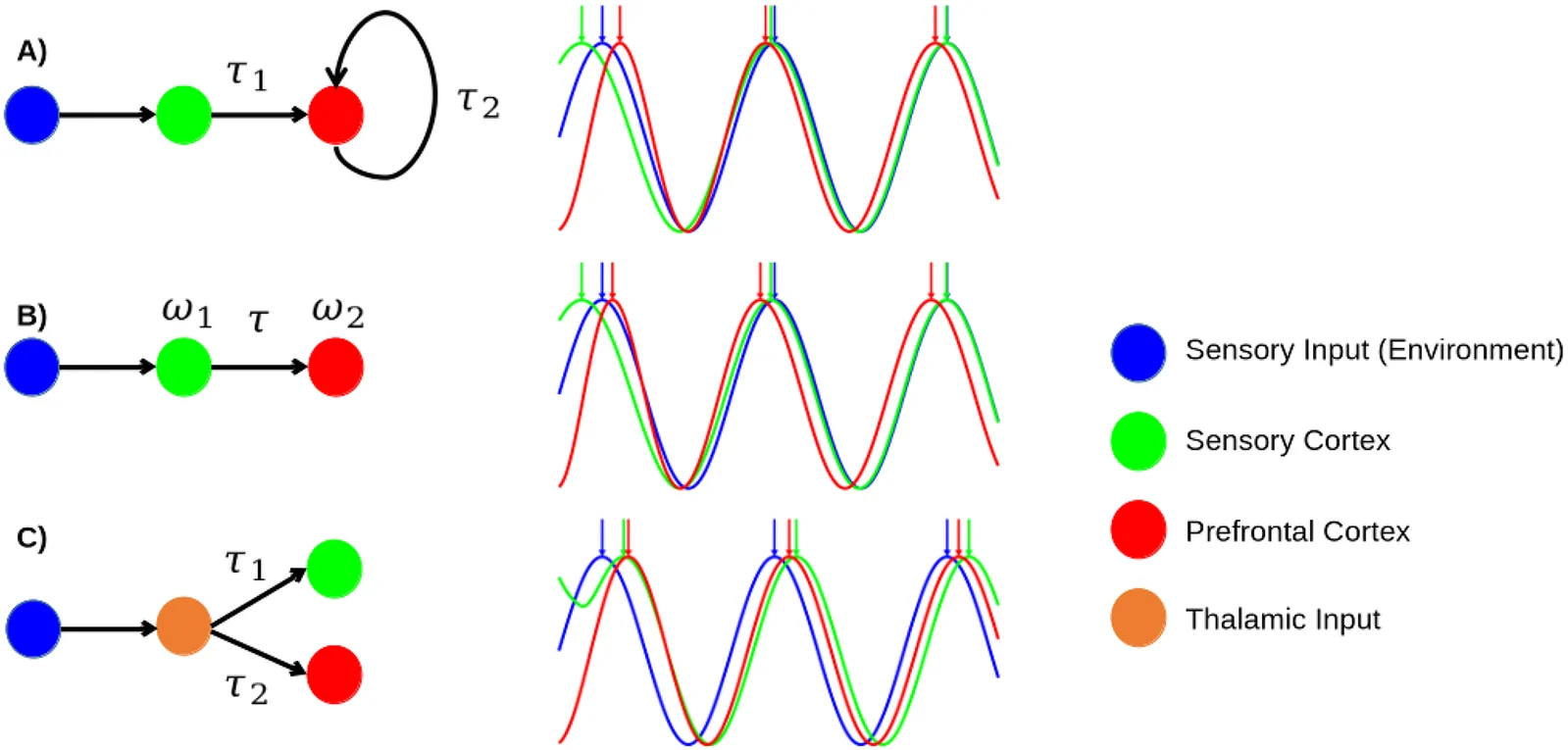
Proposed solutions that meet all the temporal constraints. The plots are sinusoids of phases of simulated coupled oscillators. *τ*’s represent time lags and *ω*’s represent intrinsic frequencies. ***A***, Anticipated synchronization (AS), *τ*_1_ is the transmission lag and *τ*_2_ is the delayed self-feedback. ***B***, Frequency mismatch, with *ω*_1_ < *ω*_2_ and ***C***, Thalamic input with *τ*_1_ > *τ*_2_.

But how can one impose anticipation from an oscillatory coupling perspective? One proposal is anticipated synchronization (AS; Voss, 2000). AS is an unusual phenomenon in dynamical systems that emerges when a receiver starts anticipating the sender’s future activity. This seemingly paradoxical coupling is possible through the addition of a delayed inhibitory feedback loop in the receiver system (a trace of its own history). Interestingly, this happens in a unidirectional coupling scheme (feedforward). Since this scheme is unidirectional from sensory input to PFC (via SC), the SC can remain strongly coupled to the sensory input. It can be seen in Figure 4*A* how the excitable phase of PFC starts leading the phase of SC in anticipation. Through this anticipation, information can be sent from PFC back to SC, arriving at a future moment in time corresponding to the optimal phase, while SC remains coupled to the sensory input. Thus, AS satisfies all the temporal constraints. Note that, in this context, “anticipation” refers to anticipation of oscillations rather than the conscious effort of anticipation.

**Figure 5.**
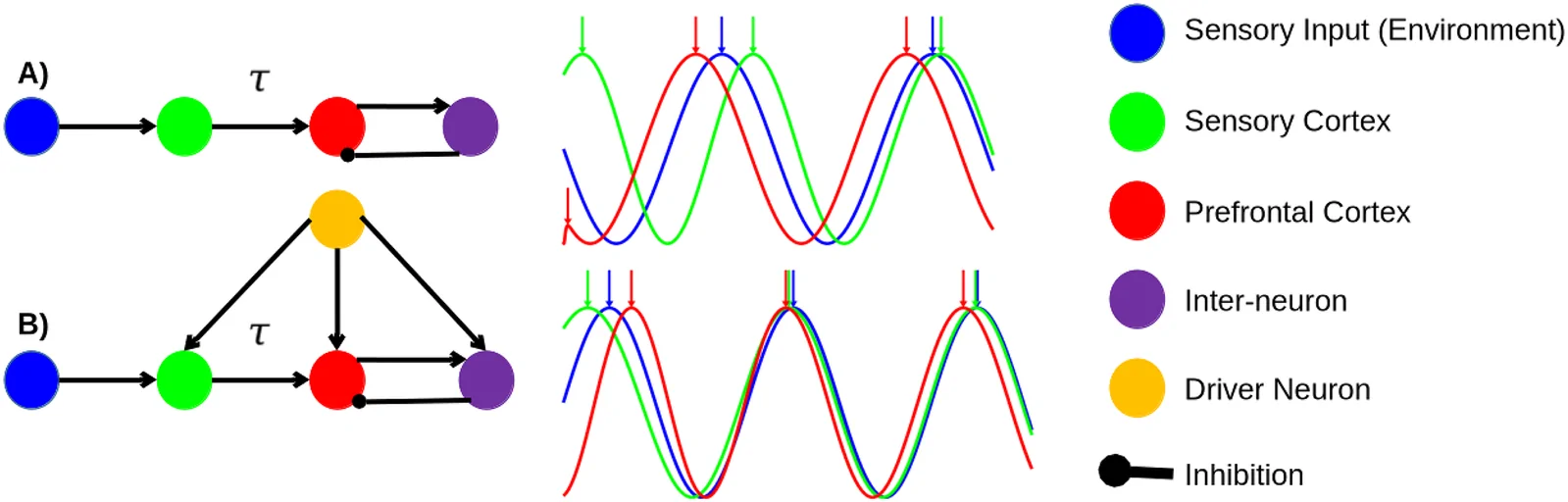
Anticipated synchronization through biologically plausible motifs based on Matias et al. (2011). ***A***, is the model without a driver neuron and ***B***, is the model with a driver neuron.

AS has been demonstrated and studied in several biologically plausible neuronal models and motifs (Toral et al., 2003; Ciszak et al., 2004; Matias et al., 2011, 2016, 2017). AS has also been demonstrated in behavioral studies (Stepp, 2009; Washburn et al., 2015) and also via EEG (electroencephalogram) recordings (Carlos et al., 2020). Additionally, the inhibitory autapse (self-inhibitory feedback synapse) in the brain could be part of potential feedback loops (Pinto et al., 2019; Ding et al., 2023). However, it is still unknown how the exact mechanism of the internal PFC loop could work. Are AS feedback loops implemented locally or rather follow a long-range connection? (Matias et al., 2011) propose biologically plausible mechanisms through an inhibitory feedback loop from an inter-neuron with and without a driver neuron (Fig. 5*B* and *A* respectively). In the original work (Matias et al., 2011), the authors model these motifs with a single neuron model (Hodgkin–Huxley model), and without any constraint of sensory input. Here, we adapted these models to be similar to the Stuart–Landau oscillators as in Figure 4, but added the inter-neuron and driver nodes (see github link for details). Both these AS implementations show that the SC couples with the sensory input and the PFC acts ahead of the SC in a feedforward motif, thus satisfying all three constraints (Fig. 5). In sum, AS is a viable solution for the temporal feedback-feedforward paradox, but more evidence is needed to support the presence of these mechanisms in the brain.

## Motif #2: Frequency Mismatches Create Phase Lags

Another possible solution to this temporal dilemma is to impose frequency mismatches between SC and PFC. If PFC operates at a slightly higher-frequency, it can act before SC. Oscillatory coupling dynamics manifest such that an oscillator always pulls toward its natural frequency. When a PFC oscillator is (weakly) coupled to a SC oscillator at a slower frequency compared to its own natural frequency, the PFC oscillator is pulled to this slower one and at high enough coupling strengths will follow the frequency of the slower oscillator. However, due to its internal dynamics being faster and the additional pull to its own natural frequency, PFC will actually lead in phase (even though it is coupled in a unidirectional feedforward manner). Although information transfer from SC to PFC will happen at a slower rate (compared to PFC’s intrinsic frequency), the phase of PFC will still be ahead of SC due to its faster intrinsic frequency. In our case, the oscillatory motif and the model for this scheme are identical to the purely feedforward scheme (Fig. 3*B*), except that we assume that the natural frequency of the PFC is slightly greater than the SC. It can be seen from Figure 4*B* that the PFC’s excitable phases (peaks) act a bit before SC’s excitable phases, leaving PFC enough time for providing contextual information. Also, the SC couples with the sensory input. This satisfies all the three constraints.

The plausibility of frequency mismatches has been suggested by Lowet et al. (2022). They propose that slight mismatches in the frequencies (detuning) of different regions are not only important for phase-locking but also for establishing preferred phase relations between the oscillators, and thereby preferred communication directions. However, Lowet et al. (2022) have shown this detuning of frequencies in the gamma domain. The natural frequency in the low-frequency domain is still debated in which some report a decrease in frequency across the cortical hierarchy (Keitel and Gross, 2016) and others an increase (Capilla et al., 2022). It is an open question whether frequency detuning could be a mechanism for neuronal communication scaffolding in the theta range considering these inconsistencies in reported frequency shifts across the cortical hierarchy.

## Motif #3: Common Input, Different Lags

A final solution is that the SC and PFC are dynamically controlled by a common input. Owing to the temporal constraints, common input must relay the information to PFC through a faster route (smaller transmission lag) compared to SC. In this way, the phase of the PFC will reach an excitable state before the excitable phase of SC (Fig. 4*C*). In this scheme, the neuronal communication scaffold is not achieved by direct coupling between regions, but rather indirectly governed by a common input at different delays. Note that, in the previously discussed feedforward motifs, we assume no time lag between sensory input and SC. Therefore, the SC couples and synchronizes perfectly with the sensory input. However, in this case the SC is coupled with the thalamic input. Due to the presence of time delay, the sensory cortex is lagged but still locked to the sensory input, thus satisfying all the constraints.

The most likely common input to a wide variety of cortical areas is the thalamus, which has been shown to relay information to multiple cortical areas (Guillery and Sherman, 2002; Halassa and Kastner, 2017). Thalamus can relay information to different brain areas via different transmission lags. This kind of coupling scheme could be imagined in the visual system, as it is known that thalamus relays information either through a faster (magnocellular) or a slower (parvocellular) pathway (Nowak et al., 1995; Jeantet et al., 2018; Atilgan et al., 2020; Wang et al., 2023), which would fit if the magnocellular pathway links to neuronal scaffolding. However, there is no evidence that these two separate pathways relate to neuronal scaffolding in the theta domain. Additionally, it is unclear whether these dual routes also exist in other sensory modalities. The pulvinar (the largest thalamic nucleus) has also been shown to orchestrate synchronization of different cortical areas, thus facilitating the transmission of information. Additionally, the claustrum could act as a common input, as it has been shown to not only integrate widespread cortical responses (Shelton et al., 2025) but also support long-range projections to different brain areas (Lei et al., 2025).

## Concluding Remarks

This article explores a fundamental dilemma of temporal processing: How can the SC process the sensory input while simultaneously receiving top-down feedback from the PFC? Here, we address this challenge by highlighting temporal constraints that should be accounted for to enable efficient communication in the framework of coupled oscillators. An optimal coupling scheme should maintain the coupling of SC to the sensory input, account for transmission delays and align information transfer to highly excitable phases. From a dynamical systems view, the low-frequency scaffolding from SC to PFC has to be feedforward for optimal communication. Any simultaneous feedback coupling from PFC to SC will make SC decouple from the sensory input and therefore will not work as an optimal communication scheme.

Here, we investigated how low-frequency oscillations can provide a scaffold for information processing, suggested, for example, in Fiebelkorn et al. (2018) and Mohan et al. (2024). While the theta band plays a prominent role in long-range communication, a similar coupling could also involve the alpha band (Michalareas et al., 2016; Solís-Vivanco et al., 2018). All proposed temporal constraints and mechanisms in this manuscript are not necessarily specific to theta and can easily be generalized to other frequency bands that provide neural scaffolding. We propose a mechanism in which information flows bidirectionally along a scaffold of highly excitable theta phases, which provide temporal windows of opportunity. Within this low-frequency scaffold, higher-frequency oscillations likely carry the information content (Box 2). Simultaneous bidirectional scaffolding does not satisfy all temporal constraints needed for optimal communication as demonstrated using coupled oscillators. (The code and mathematical description of all the models can be found here: github link has been redacted for double-blind peer review process. The code and mathematical derivations are attached separately.) Hence, we propose unidirectional feedforward coupling mechanisms, either through AS, frequency mismatch, or common input, as solutions to this temporal dilemma. Through coupled oscillators, we show how these schemes offer viable dynamical solutions that satisfy all the temporal constraints. Across all three proposed mechanisms, empirical evidence remains limited, reflecting the lack of causal modeling approaches that are directly integrated with empirical data. The frequency mismatch hypothesis is inconsistent with evidence suggesting lower, rather than higher, frequencies in higher-order cortical regions (Keitel and Gross, 2016). Common input mechanisms could leverage thalamic output, but this is not empirically linked to low-frequency scaffolding across distributed sites. Moreover, most connections are reported to arrive earlier in sensory compared to frontal areas (Poghosyan and Ioannides, 2008). AS mechanisms are consistent with findings regarding a leading PFC phase, but empirical evidence is limited and mostly pertains to the motor system (Washburn et al., 2015; Roman et al., 2019). Moreover, the precise nature and neural implementation of the required feedback loops remain to be clarified.

We do not claim that these solutions are the only possible solutions, but we highlight that any other solution within the framework of coupled oscillators fulfilling all temporal constraints posed here must logically be feedforward. Any other alternative for neuronal communication scaffolding must accept that the brain does not always process information optimally. If so, future proposals including feedback must be explicit to which temporal constraint is dropped. Does the brain sometimes communicate on non-optimal phases (dropping constraint #1, continuous input tracking)? For example, reduced oscillatory power may broaden information processing windows (Hanslmayr et al., 2012; Jensen et al., 2012), potentially reducing the importance of precise alignment with peak excitability phases (constraint #1). If so, one would expect that power is more desynchronized when feedforward and feedback processing occur concurrently compared to isolated feedforward and feedback events. Or does the brain decouple dynamically from the environment (dropping constraint #3, continuous input tracking)? Alternative frameworks outside the domain of phase-coupled oscillators have also been proposed, however addressing all these approaches is beyond the scope of the present work (Jacobs et al., 2007; Lee et al., 2013; Larkum et al., 2018; Mikulasch et al., 2023).

The motifs we propose assume a simple periodic input of a sine wave, which conceptually represents the temporal structure of the environment. However, these motifs can also function without such a sine wave but with other types of temporal structure. For example, AS has been shown to work for many other types of chaotic systems that, at first glance, do not have a clear temporal structure (Voss, 2000; Masoller and Zanette, 2001). However, the dynamical system can still anticipate the response. Additionally, it has also been suggested that environmental processing occurs through internally generated active sampling (Parr et al., 2022). This mechanism would not exhibit the alignment issue we propose, as the SC effectively decouples from the environment (constraint #3). Such a system will track temporal structure in the environment when a top-down sampling plan of the predicted timing of input would align the SC to the environment rather than using bottom-up entrainment mechanisms (Obleser and Kayser, 2019; Kaltenmaier et al., 2025). This system has low input coupling (loosening constraint #3) but could empirically result in a spurious coupling between the environment and SC since the timing of low-frequency oscillations is a result of the accurate sampling. The current work is grounded in the assumption of the role of entrainment for sensory processing (Obleser and Kayser, 2019) and what temporal constraints follow if such a mechanism is adapted. However, we are also aware of the criticism of this account (Nandi et al., 2019; Vinck et al., 2023; Lalor and Nidiffer, 2025; Atanasova et al., 2026).

Note that we assume the role of oscillations in cross-region communication as well as its role for sensory tracking by means of neural entrainment to environmental input. However, the assumption of an oscillatory framework, and especially its role in communication, remains actively debated. In particular, Schneider et al. (2021) argue that commonly assumed signatures of cross-region communication, such as coherence, are merely a byproduct of anatomical connectivity, and are not necessarily the mechanisms of communication. Furthermore, in speech processing, the evidence for endogenous rhythms is highly debated, and it is asserted that cortical tracking may reflect transient evoked responses rather than oscillatory entrainment (Nandi et al., 2019; Lalor and Nidiffer, 2025; Atanasova et al., 2026, but also see Zoefel et al., 2018; Doelling and Assaneo, 2021). We also assume that the scaffolding of long-range communication is tied to low-frequency oscillations, and in particular to the theta band. This view also remains debated. It has been suggested that scaffolding can be tied to high frequencies that in turn drive the lower frequencies (Nandi et al., 2019). In addition, Popov et al. (2018) report that the interaction between theta and alpha can be parallel and bidirectional, with alternating regimes of dominance. However, the temporal constraints and models proposed here still hold, irrespective of the frequency band involved. Importantly, we do not claim to have solved all these controversies and know the true underlying mechanisms. Instead, we ask what temporal constraints would follow if a framework of entrainment and low-frequency scaffolding is adopted and, given these assumptions, evaluate the likely mechanisms for inter-regional brain communication.

Understanding how the brain resolves this temporal bottleneck would offer a fresh perspective on inter-area brain communication and uncover fundamental principles of cognition. Although the proposed solutions need empirical validation, they offer a theoretical framework to guide future experimental work to study the timing and directionality of inter-area communication. One first important step is to investigate connectivity in a temporally dynamic context in which feedforward and feedback are needed simultaneously, rather than an experimental environment where feedback and feedforward processes are separated or input is static. If our proposal is correct, long-range connectivity should follow a feedforward direction even though the phase of the frontal cortex is leading the phase of the SC. Note that a phase-lead of frontal cortex has been reported (Osorio and Assaneo, 2025), but in most cases this phase-lead has been inferred as reflecting feedback connectivity; we would like to stress that our models clearly show that leads or lags in phases do not equate a leading or lagging neural direction. To dissociate the motifs we propose, one needs to go one step further. The current models could be expanded to predict neural responses in situations with varying temporal dynamics and empirically validated using electrocortical data from both the sensory and frontal regions. Anticipated synchronization could be imposed using closed-loop electrical stimulation (Soleimani et al., 2023; Colucci et al., 2025). Frequency mismatches could be validated by demonstrating frequency-specific tuning during feedforward–feedback interactions in the sensory and frontal cortex. The preferred frequency of individual regions could be extracted by validating Arnold tongue-like tongue behavior (stronger coupling for frequencies close to its internal frequency). Validating common input is the most difficult, as in the ideal scenario one needs access to three different regions. However, if this is feasible, coupling dynamics and transmission delays of this motif can be tested.

In this work we focused on SC and PFC; however, these solutions and the temporal constraints can also be relevant for communication of other brain regions. Sensory regions can process feedback information from multiple regions within the SC and beyond (Bastos et al., 2015a; Michalareas et al., 2016). Understanding the temporal dynamics of such complex motifs remains a challenge. The models proposed here are mainly focused on pair-wise interaction. Processing higher-order interaction would require the model to not only satisfy the temporal constraints but also account for the timing and dynamic routing of information. Multiple feedback connections would be subject to heterogeneous delays and need to be orchestrated in a way that they converge to the same excitable window of a sensory region. This could potentially require weighted integration of feedback information depending on the cognitive context/task. We highlight that it is essential to view the brain as a flexible and dynamic system in which most regions need to process feedforward and feedback information at the same time. Empirical findings of separate feedforward and feedback directionality are likely a consequence of study designs that experimentally separate feedforward and feedback processes, for example in working memory studies showing separation between encoding and retrieval (Mohsenzadeh et al., 2018; Kim, 2019), or studies with static images (Mohsenzadeh et al., 2018; Motlagh et al., 2024). We believe such situations do not reflect the default of brain processing, which rather keeps on processing incoming input while updating beliefs about the external world according to neural feedback.

10.1523/ENEURO.0012-26.2026.d1 Data 1 Download Data 1, ZIP file.

## Synthesis

Reviewing Editor: Niko Busch, Westfalische Wilhelms-Universitat Munster Decisions are customarily a result of the Reviewing Editor and the peer reviewers coming together and discussing their recommendations until a consensus is reached. When revisions are invited, a fact-based synthesis statement explaining their decision and outlining what is needed to prepare a revision will be listed below. The following reviewer(s) agreed to reveal their identity: Mathilde Bonnefond, Anne Keitel.

# Synthesis

Thank you for submitting your manuscript entitled "I can't hear you both at the same time: A temporal dilemma during inter-regional communication." The reviewers find that the manuscript addresses an interesting and important theoretical question about how feedforward and feedback interactions can be coordinated under temporal constraints. Both reviewers appreciate the conceptual framework and the effort to formalize the problem within an oscillatory coupling perspective.

At the same time, the reviewers raise several points that should be addressed in a revision. In particular, they encourage you to broaden the discussion of relevant literature and assumptions underlying the framework. This includes discussing findings that may challenge or complicate oscillatory accounts of inter-regional communication, clarifying several aspects of the proposed mechanisms and constraints, and expanding the discussion of possible empirical tests of the proposed model.

If you decide to submit a revised manuscript, please provide a detailed point-by-point response to the reviewers' comments.

# Reviewer 1

The paper titled "I can't hear you both at the same time: A temporal dilemma during inter-regional communication" addresses the challenge of integrating sensory input with feedback from higher-order brain regions, such as the prefrontal cortex (PFC). The authors propose three key constraints for optimal inter-area communication. First, information should arrive at the most excitable phase of a receiving group. Second, transmission delays must be accounted for. Third, the sensory cortex (SC) must remain coupled to the sensory input. To address these constraints, the paper suggests three motifs. The first motif relies on Anticipated Synchronization (AS) through inhibitory loops within the PFC. The second motif is based on the possibility of frequency mismatches between the SC and PFC. The third motif involves a common input with different lags.

I think this paper addresses a very important issue regarding the temporal integration of feedforward and feedback communication. It is an opinion paper with which we can disagree, but it explicitly states that it does not aim to comprehensively suggest all solutions to this problem of integration. Instead, it limits itself to an oscillatory-based framework and focuses on optimal processing solutions. It also emphasizes the need for experimental validation.

I have a few comments that I would like the authors to address:

1. The paper primarily focuses on feedback from the PFC to the SC. However, sensory regions receive feedback from multiple regions within the sensory hierarchy and beyond (e.g., Michalareas et al., 2016; Bastos et al., 2015). Future models could incorporate this complexity to provide a more comprehensive understanding of inter-regional communication. Meanwhile, I believe this issue should be addressed in the text.

2. Somehow related to the second motif, power changes in different regions could affect information processing windows in addition to or instead of frequency differences. For instance, a decrease in low-frequency power might increase the window of information processing in the SC. I suggest the authors briefly discuss how this factor, power change, could influence their model.

3. Related to the third motif, the claustrum and pulvinar (a specific nucleus of the thalamus) are highly connected regions with different levels of specificity and extent that could play a role in orchestrating interactions between regions and could be mentioned.

4. The paper assumes that the SC should follow the temporal constraints of the sensory input, which is represented here as a sinusoidal shape (which should be further discussed in itself). Even though the authors mention they focus on mechanisms that would underlie optimal processing, I believe it is worth discussing more that the brain might sample information based on an internal clock, perhaps not optimally as many results seem to indicate (e.g. Marti et al. 2012), or through active sampling of information via ocular or motor changes (Schroeder et al., 2019; Parr et al., 2022). This could be discussed in the context of the paper's assumptions about sensory input processing.

5. Although theta is a valuable frequency for PFC-SC interaction, many papers indicate that this interaction could occur within the alpha-band (e.g., Michalareas et al., 2016; Solis-Vivanco et al., 2018). It does not change the overall reasoning but could be mentioned.

6. The paper acknowledges the need for experimental validation. More specific experimental approaches, together with specific predictions, could be suggested to test the proposed motifs.

7. The paper uses computational models to explore the proposed motifs. As the link to Github was not available due to the reviewing process, I could not explore these models. However, it might be worth discussing how these models could be expanded or refined to include more biological details.

References

• Bastos, A.M., Vezoli, J., Bosman, C.A., Schoffelen, J.M., Oostenveld, R., Dowdall, J.R., De Weerd, P., Kennedy, H., Fries, P. (2015). Visual areas exert feedforward and feedback influences through distinct frequency channels. Neuron, 85(2), 390-401.

• Dillingham CM, Jankowski MM, Chandra R, Frost BE, O'Mara SM. (2017) The claustrum: Considerations regarding its anatomy, functions and a programme for research. Brain Neurosci Adv. 13;1:2398212817718962

• Michalareas, G., Vezoli, J., Van Pelt, S., Schoffelen, J.M., Kennedy, H., Fries, P. (2016). Alpha-beta and gamma rhythms subserve feedback and feedforward influences among human visual cortical areas. Neuron, 89(2), 384-397.

• Marti, S, Sigman M., Dehaene S. (2012) A shared cortical bottleneck underlying Attentional and psychological refracrory Period. Neuroimage. 1;59(3):2883-98

• Parr, Pezzulo, Friston (2022) The free Energy Principle In Mind, Brain and Behavior. MIT Press.

• Saalmann Y.R., Pinsk M.A., Wang L., Li X, Kastner S. (2012) The Pulvinar Regulates Information Transmission Between Cortical Areas Based on Attention Demands. Science 337,753-756

• Schroeder, C.E., Lakatos, P. (2009). Low-frequency neuronal oscillations as instruments of sensory selection. Trends in Neurosciences, 32(1), 9-18.

• Solis-Vivanco R., Jensen O., Bonnefond M. (2018) Top-down Control Of Alpha Phase Adjustment In Anticipation of temporally Predictable Visual Stimuli. Journal of Cognitive Neuroscience (2018) 30 (8): 1157-1169.

# Reviewer 2

This is an interesting and timely manuscript that can be followed well despite the modelling-heavy content. I have reviewed a previous version of this manuscript for a different journal, and some of the concerns have been addressed already. Below are my remaining comments.

1) To thoroughly be able to derive the constraints and propose models that meet the constraints, it would be necessary to not just cite confirming research but also critically discuss the null findings and contentious nature of oscillatory theories and findings. For example, entrainment seems to be described and taken at face value, although there is an ongoing lively debate about the mechanistic nature of cortical tracking (just two recent examples are Lalor &Nidiffer, 2025 and Atanasova et al., 2026). Further, for all findings that confirm a role of theta, beta or gamma, there are findings that don't fit, for example indicating other bands or not finding a relationship. For example, the idea that low-frequency oscillations provide a temporal scaffolding is less clear than suggested here, as sometimes it is the high frequency that provides the scaffolding/drives the low frequency (e.g. Nandi et al., 2019), and sometimes it's a bidirectional relationship between low and high frequencies (Popov et al., 2018). Overall, I think for all assumed mechanisms and constraints it is necessary to be a bit more critical to give a complete picture, instead of picking only providing evidence.

2) Would it be possible to evaluate the three suggested solutions in terms of which one is more likely, or which could happen in specific circumstances? Or related, how it would be possible to disentangle between the three suggested possibilities?

3) The authors mention "A further proposal posits that feedforward and feedback inputs are anatomically segregated within single neurons, with basal dendrites receiving feedforward input and apical tufts receiving feedback" (Box 1). Could you elaborate more on how this does not already solve the proposed paradox? If single neurons can simultaneously couple to the input and the PFC, then the problem would be solved through this mechanism?

4) Similarly, just before this it is said that "precludes precise temporal convergence, as signals carried in different frequency bands cannot achieve sustained phase alignment". If the different frequencies are nested (i.e. cross-frequency multiplexing) then there could be sustained phase alignment, so it would be good to explain why multiplexing wouldn't solve the proposed paradox.

5) When constraint numbers are mentioned after they have been introduced, it would be helpful if it was briefly mentioned again what they were, as otherwise the reader has to go back to remind themselves of the content (a short label occasionally would help with this).

6) Line 226: "the inhibitory autapse" - could you elaborate what that means?

7) Line 279: "the sensory cortex is lagged but still locked to the sensory input (no delay between sensory input and SC)" How can there be a lag but no delay at the same time? Do the authors ascribe different meanings to "lag" and "delay"?

8) The manuscript contains a lot of grammatical errors/typos that were a bit distracting. It would be great if it could be carefully proofread before resubmitting. For example: "several temporal constraints such phase excitability", "How can one brain region can simultaneously", "Electrodes arrays".

References

Atanasova, T., Gross, J., Rimmele, J. M., &Keitel, A. (2026). The involvement of endogenous brain rhythms in speech processing. Neuroscience &Biobehavioral Reviews, 106568.

Lalor, E., &Nidiffer, A. (2025). On the generative mechanisms underlying the cortical tracking of natural speech: a position paper. OSF Preprints.

Nandi, B., Swiatek, P., Kocsis, B., &Ding, M. (2019). Inferring the direction of rhythmic neural transmission via inter-regional phase-amplitude coupling (ir-PAC). Sci Rep, 9(1), 6933.

Popov, T., Popova, P., Harkotte, M., Awiszus, B., Rockstroh, B., &Miller, G. A. (2018). Cross-frequency interactions between frontal theta and posterior alpha control mechanisms foster working memory. NeuroImage, 181, 728-733.
