# Supplementary material for: I Can’t Hear You Both at the Same Time: A Temporal Dilemma During Inter-Regional Communication
Source: eNeuro. 2026 Aug 4;13(8):ENEURO.0012-26.2026. doi: 10.1523/ENEURO.0012-26.2026 (PMC13555238; doi:10.1523/ENEURO.0012-26.2026)
Supplement: Supplementary file 1 [file eneuro-13-ENEURO.0012-26.2026-s001.zip › CommunicationDilemma_models-main/model_derivations.pdf]

1 Phase Reductions

For all the phase reductions we assume following polar forms :

$$I(t) = e^{i\theta_I(t)} \tag{1}$$

$$S = e^{i\theta_S} \tag{2}$$

$$F = e^{i\theta_F} \tag{3}$$

With the derivatives as:

$$\dot{S} = i\dot{\theta}_S e^{i\theta_S} \tag{4}$$

$$\dot{F} = i\dot{\theta}_F e^{i\theta_F} \tag{5}$$

1.1 Anticipated Synchronization

Model Equations

Consider the oscillators S and F described by:

$$\dot{S} = S(\alpha_S - |S|^2) + i\beta_S S|S|^2 + k(I(t) - S) \tag{6}$$

$$\dot{F} = F(\alpha_F - |F|^2) + i\beta_F F|F|^2 + \epsilon(S - F(t-\tau)) \tag{7}$$

Equating Real and Imaginary Parts for S

Equating the derivatives from the model equations and the polar form:

$$i\dot{\theta}_S e^{i\theta_S} = e^{i\theta_S}(\alpha_S - 1) + i\beta e^{i\theta_S} + k(e^{i\theta_I(t)} - e^{i\theta_S}) \tag{8}$$

Dividing both sides by $e^{i\theta_S}$:

$$i\dot{\theta}_S = (\alpha_S - 1) + i\beta_S + k(e^{i(\theta_I(t)-\theta_S)} - 1) \tag{9}$$

Using Euler's formula $e^{i(\theta_I(t)-\theta_S)} = \cos(\theta_I(t) - \theta_S) + i\sin(\theta_I(t) - \theta_S)$:

$$\begin{aligned} i\dot{\theta}_S = &(\alpha_S - 1) + i\beta_S \\ &+ k(\cos(\theta_I(t) - \theta_S) + i\sin(\theta_I(t) - \theta_S) - 1) \end{aligned} \tag{10}$$

Separating into real and imaginary parts:

$$0 = (\alpha_S - 1) + k(\cos(\theta_I(t) - \theta_S) - 1) \tag{11}$$

$$\dot{\theta}_S = \beta_S + k\sin(\theta_I(t) - \theta_S) \tag{12}$$

Equating Real and Imaginary Parts for F

Equating the derivatives from the model equations and the polar form:

$$i\dot{\theta}_F e^{i\theta_F} = e^{i\theta_F}(\alpha_F - 1) + i\beta_F e^{i\theta_F} + \epsilon(e^{i\theta_S} - e^{i\theta_F(t-\tau)}) \tag{13}$$

Dividing both sides by $e^{i\theta_F}$:

$$i\dot{\theta}_F = (\alpha_F - 1) + i\beta_F + \epsilon(e^{i(\theta_S-\theta_F)} - e^{i(\theta_F(t-\tau)-\theta_F)}) \tag{14}$$

Using Euler's formula $e^{i(\theta_S-\theta_F)} = \cos(\theta_S - \theta_F) + i\sin(\theta_S - \theta_F)$:

$$i\dot{\theta}_F = (\alpha_F - 1) + i\beta_F + \epsilon\left[(\cos(\theta_S - \theta_F) + i\sin(\theta_S - \theta_F)) - e^{i(\theta_F(t-\tau)-\theta_F)}\right] \tag{15}$$

Separating into real and imaginary parts:

$$0 = (\alpha_F - 1) + \epsilon(\cos(\theta_S - \theta_F) - \cos(\theta_F(t-\tau) - \theta_F)) \tag{16}$$

$$\dot{\theta}_F = \beta_F + \epsilon\left(\sin(\theta_S - \theta_F) - \sin(\theta_F(t-\tau) - \theta_F)\right) \tag{17}$$

Phase Reduced Model

$$\dot{\theta}_S = \beta_S + k\sin(\theta_I(t) - \theta_S) \tag{18}$$

$$\dot{\theta}_F = \beta_F + \epsilon(\sin(\theta_S - \theta_F) - \sin(\theta_F(t-\tau) - \theta_F)) \tag{19}$$

1.2 Feedforward-Feedback Coupling

Model Equations

Consider the oscillators S and F described by:

$$\dot{S} = S(\alpha_S - |S|^2) + i\beta_S S|S|^2 + k(I(t) - S) + \epsilon(F(t-\tau) - S) \tag{20}$$

$$\dot{F} = F(\alpha_F - |F|^2) + i\beta_F F|F|^2 \tag{21}$$

Equating Real and Imaginary Parts for S

Equating the derivatives from the model equations and the polar form:

$$i\dot{\theta}_S e^{i\theta_S} = e^{i\theta_S}(\alpha_S - 1) + i\beta_S e^{i\theta_S} + k(e^{i\theta_I(t)} - e^{i\theta_S}) + \epsilon(e^{i\theta_F(t-\tau)} - e^{i\theta_S}) \tag{22}$$

Dividing both sides by $e^{i\theta_S}$:

$$i\dot{\theta}_S = (\alpha_S - 1) + i\beta_S + k(e^{i(\theta_I(t)-\theta_S)} - 1) + \epsilon(e^{i(\theta_F(t-\tau)-\theta_S)} - 1) \tag{23}$$

Using Euler's formula $e^{i(\theta_I(t)-\theta_S)} = \cos(\theta_I(t) - \theta_S) + i\sin(\theta_I(t) - \theta_S)$:

$$\begin{aligned} i\dot{\theta}_S = &(\alpha_S - 1) + i\beta_S \\ &+ k(\cos(\theta_I(t) - \theta_S) + i\sin(\theta_I(t) - \theta_S) - 1) \\ &+ \epsilon(\cos(\theta_F(t-\tau) - \theta_S) + i\sin(\theta_F(t-\tau) - \theta_S) - 1) \end{aligned} \tag{24}$$

Separating into real and imaginary parts:

$$0 = (\alpha_S - 1) + k(\cos(\theta_I(t) - \theta_S) - 1) + \epsilon(\cos(\theta_F(t-\tau) - \theta_S) - 1) \tag{25}$$

$$\dot{\theta}_S = \beta_S + k\sin(\theta_I(t) - \theta_S) + \epsilon\sin(\theta_F(t-\tau) - \theta_S) \tag{26}$$

Equating Real and Imaginary Parts for F

Equating the derivatives from the model equations and the polar form:

$$i\dot{\theta}_F e^{i\theta_F} = e^{i\theta_F}(\alpha_F - 1) + i\beta_F e^{i\theta_F} \tag{27}$$

Dividing both sides by $e^{i\theta_F}$:

$$i\dot{\theta}_F = (\alpha_F - 1) + i\beta_F \tag{28}$$

Separating into real and imaginary parts:

$$0 = (\alpha_F - 1) \tag{29}$$

$$\dot{\theta}_F = \beta_F \tag{30}$$

Phase Reduced Model

$$\dot{\theta}_S = \beta_S + k\sin(\theta_I(t) - \theta_S) + \epsilon\sin(\theta_F(t-\tau) - \theta_S) \tag{31}$$

$$\dot{\theta}_F = \beta_F \tag{32}$$

1.3 Purely Feedforward Coupling

Model Equations

Consider the oscillators S and F described by:

$$\dot{S} = S(\alpha_S - |S|^2) + i\beta_S S|S|^2 + k(I(t) - S) \tag{33}$$

$$\dot{F} = F(\alpha_F - |F|^2) + i\beta_F F|F|^2 + \epsilon(S(t-\tau) - F) \tag{34}$$

Equating Real and Imaginary Parts for S

Equating the derivatives from the model equations and the polar form:

$$i\dot{\theta}_S e^{i\theta_S} = e^{i\theta_S}(\alpha_S - 1) + i\beta_S e^{i\theta_S} + k(e^{i\theta_I(t)} - e^{i\theta_S}) \tag{35}$$

Dividing both sides by $e^{i\theta_S}$:

$$i\dot{\theta}_S = (\alpha_S - 1) + i\beta_S + k(e^{i(\theta_I(t)-\theta_S)} - 1) \tag{36}$$

Using Euler's formula $e^{i(\theta_I(t)-\theta_S)} = \cos(\theta_I(t) - \theta_S) + i\sin(\theta_I(t) - \theta_S)$:

$$\begin{aligned} i\dot{\theta}_S = {} & (\alpha_S - 1) + i\beta_S \\ & + k(\cos(\theta_I(t) - \theta_S) + i\sin(\theta_I(t) - \theta_S) - 1) \end{aligned} \tag{37}$$

Separating into real and imaginary parts:

$$0 = (\alpha_S - 1) + k(\cos(\theta_I(t) - \theta_S) - 1) \tag{38}$$

$$\dot{\theta}_S = \beta_S + k\sin(\theta_I(t) - \theta_S) \tag{39}$$

Equating Real and Imaginary Parts for F

Equating the derivatives from the model equations and the polar form:

$$\begin{aligned} i\dot{\theta}_F e^{i\theta_F} = {} & e^{i\theta_F}(\alpha_F - 1) + i\beta_F e^{i\theta_F} \\ & + \epsilon(e^{i\theta_S(t-\tau)} - e^{i\theta_F}) \end{aligned} \tag{40}$$

Dividing both sides by $e^{i\theta_F}$:

$$i\dot{\theta}_F = (\alpha_F - 1) + i\beta_F + \epsilon(e^{i(\theta_S(t-\tau)-\theta_F)} - 1) \tag{41}$$

Using Euler's formula $e^{i(\theta_S(t-\tau)-\theta_F)} = \cos(\theta_S(t-\tau) - \theta_F) + i\sin(\theta_S(t-\tau) - \theta_F)$:

$$\begin{aligned} i\dot{\theta}_F = {} & (\alpha_F - 1) + i\beta_F \\ & + \epsilon(\cos(\theta_S(t-\tau) - \theta_F) + i\sin(\theta_S(t-\tau) - \theta_F) - 1) \end{aligned} \tag{42}$$

Separating into real and imaginary parts:

$$0 = (\alpha_F - 1) + \epsilon(\cos(\theta_S(t-\tau) - \theta_F) - 1) \tag{43}$$

$$\dot{\theta}_F = \beta_F + \epsilon\sin(\theta_S(t-\tau) - \theta_F) \tag{44}$$

Phase Reduced Model

$$\dot{\theta}_S = \beta_S + k\sin(\theta_I(t) - \theta_S) \tag{45}$$

$$\dot{\theta}_F = \beta_F + \epsilon\sin(\theta_S(t-\tau) - \theta_F) \tag{46}$$

1.4 Thalamic Input

Model Equations

Consider the oscillators S and F described by:

$$\dot{S} = S(\alpha_S - |S|^2) + i\beta_S S|S|^2 + k(I(t - \tau_1) - S) \tag{47}$$

$$\dot{F} = F(\alpha_F - |F|^2) + i\beta_F F|F|^2 + \epsilon(I(t - \tau_2) - F) \tag{48}$$

Equating Real and Imaginary Parts for S

Equating the derivatives from the model equations and the polar form:

$$i\dot{\theta}_S e^{i\theta_S} = e^{i\theta_S}(\alpha_S - 1) + i\beta_S e^{i\theta_S} + k(e^{i\theta_I(t-\tau_1)} - e^{i\theta_S}) \tag{49}$$

Dividing both sides by $e^{i\theta_S}$:

$$i\dot{\theta}_S = (\alpha_S - 1) + i\beta_S + k(e^{i(\theta_I(t-\tau_1)-\theta_S)} - 1) \tag{50}$$

Using Euler's formula $e^{i(\theta_I(t-\tau_1)-\theta_S)} = \cos(\theta_I(t-\tau_1)-\theta_S)+i\sin(\theta_I(t-\tau_1)-\theta_S)$:

$$\begin{aligned} i\dot{\theta}_S = (\alpha_S - 1) + i\beta_S \\ + k(\cos(\theta_I(t - \tau_1) - \theta_S) + i\sin(\theta_I(t - \tau_1) - \theta_S) - 1) \end{aligned} \tag{51}$$

Separating into real and imaginary parts:

$$0 = (\alpha_S - 1) + k(\cos(\theta_I(t - \tau_1) - \theta_S) - 1) \tag{52}$$

$$\dot{\theta}_S = \beta_S + k\sin(\theta_I(t - \tau_1) - \theta_S) \tag{53}$$

Equating Real and Imaginary Parts for F

Equating the derivatives from the model equations and the polar form:

$$i\dot{\theta}_F e^{i\theta_F} = e^{i\theta_F}(\alpha_F - 1) + i\beta_F e^{i\theta_F} + \epsilon(e^{i\theta_I(t-\tau_2)} - e^{i\theta_F}) \tag{54}$$

Dividing both sides by $e^{i\theta_F}$:

$$i\dot{\theta}_F = (\alpha_F - 1) + i\beta_F + \epsilon(e^{i(\theta_I(t-\tau_2)-\theta_F)} - 1) \tag{55}$$

Using Euler's formula $e^{i(\theta_I(t-\tau_2)-\theta_F)} = \cos(\theta_I(t - \tau_2) - \theta_F) + i\sin(\theta_I(t - \tau_2) - \theta_F)$:

$$\begin{aligned} i\dot{\theta}_F = (\alpha_F - 1) + i\beta_F \\ + \epsilon(\cos(\theta_I(t - \tau_2) - \theta_F) + i\sin(\theta_I(t - \tau_2) - \theta_F) - 1) \end{aligned} \tag{56}$$

Separating into real and imaginary parts:

$$0 = (\alpha_F - 1) + \epsilon(\cos(\theta_I(t - \tau_2) - \theta_F) - 1) \tag{57}$$

$$\dot{\theta}_F = \beta_F + \epsilon\sin(\theta_I(t - \tau_2) - \theta_F) \tag{58}$$

Phase Reduced Model

$$\dot{\theta}_S = \beta_S + k \sin(\theta_I(t - \tau_1) - \theta_S) \tag{59}$$
$$\dot{\theta}_F = \beta_F + \epsilon \sin(\theta_I(t - \tau_2) - \theta_F) \tag{60}$$

Phase Reduced Model

$$\dot{\theta}_S = \lambda_S \left(\beta_S + k \sin(\theta_I(t) - \theta_S)\right) \tag{61}$$
$$\dot{\theta}_F = \lambda_F \left(\beta + \epsilon \sin(\theta_S(t - \tau) - \theta_F)\right) \tag{62}$$

1.5 Anticipation via inter-neuron

Model Equations

Let I (external input), S (sensory cortex), F (PFC), and N (interneuron) be Stuart-Landau oscillators:

$$\dot{S} = S(\alpha_S - |S|^2) + i\beta_S S|S|^2 + k_{IS}(I - S) \tag{63}$$
$$\dot{F} = F(\alpha_F - |F|^2) + i\beta_F F|F|^2 + k_{SF}(S - F) + k_{NF}(N - F) \tag{64}$$
$$\dot{N} = N(\alpha_N - |N|^2) + i\beta_N N|N|^2 + k_{FN}(F - N) \tag{65}$$

Assume unit amplitude: $S = e^{i\theta_S}, F = e^{i\theta_F}, N = e^{i\theta_N}, I = e^{i\theta_I}$

Equating Real and Imaginary Parts for S

$$i\dot{\theta}_S e^{i\theta_S} = e^{i\theta_S}(\alpha_S - 1) + i\beta_S e^{i\theta_S} + k_{IS}(e^{i\theta_I} - e^{i\theta_S}) \tag{66}$$

Divide by $e^{i\theta_S}$:

$$i\dot{\theta}_S = (\alpha_S - 1) + i\beta_S + k_{IS}(e^{i(\theta_I - \theta_S)} - 1) \tag{67}$$

Separate imaginary part:

$$\dot{\theta}_S = \beta_S + k_{IS} \sin(\theta_I - \theta_S) \tag{68}$$

Equating Real and Imaginary Parts for F

$$i\dot{\theta}_F e^{i\theta_F} = e^{i\theta_F}(\alpha_F - 1) + i\beta_F e^{i\theta_F} \tag{69}$$
$$+ k_{SF}(e^{i\theta_S} - e^{i\theta_F}) + k_{NF}(e^{i\theta_N} - e^{i\theta_F}) \tag{70}$$

Divide by $e^{i\theta_F}$:

$$i\dot{\theta}_F = (\alpha_F - 1) + i\beta_F + k_{SF}(e^{i(\theta_S - \theta_F)} - 1) + k_{NF}(e^{i(\theta_N - \theta_F)} - 1) \tag{71}$$

Separate imaginary part:

$$\dot{\theta}_F = \beta_F + k_{SF} \sin(\theta_S - \theta_F) + k_{NF} \sin(\theta_N - \theta_F) \tag{72}$$

Equating Real and Imaginary Parts for N

$$i\dot{\theta}_N e^{i\theta_N} = e^{i\theta_N}(\alpha_N - 1) + i\beta_N e^{i\theta_N} + k_{FN}(e^{i\theta_F} - e^{i\theta_N}) \tag{73}$$

Divide by $e^{i\theta_N}$:

$$i\dot{\theta}_N = (\alpha_N - 1) + i\beta_N + k_{FN}(e^{i(\theta_F - \theta_N)} - 1) \tag{74}$$

Separate imaginary part:

$$\dot{\theta}_N = \beta_N + k_{FN}\sin(\theta_F - \theta_N) \tag{75}$$

Phase Reduced Model

$$\dot{\theta}_I = \beta_I \tag{76}$$
$$\dot{\theta}_S = \beta_S + k_{IS}\sin(\theta_I - \theta_S) \tag{77}$$
$$\dot{\theta}_F = \beta_F + k_{SF}\sin(\theta_S - \theta_F) + k_{NF}\sin(\theta_N - \theta_F) \tag{78}$$
$$\dot{\theta}_N = \beta_N + k_{FN}\sin(\theta_F - \theta_N) \tag{79}$$

1.6 Anticipation via inter-neuron and common driver

Model Equations

Let E (external input), D (driver), M (master), S (slave), and N (interneuron) be Stuart-Landau oscillators:

$$\dot{M} = M(\alpha_M - |M|^2) + i\beta_M M|M|^2 + k_{EM}(E - M) + k_{DM}(D - M) \tag{80}$$
$$\dot{S} = S(\alpha_S - |S|^2) + i\beta_S S|S|^2 + k_{MS}(M - S) + k_{DS}(D - S) + k_{NS}(N - S) \tag{81}$$
$$\dot{N} = N(\alpha_N - |N|^2) + i\beta_N N|N|^2 + k_{SN}(S - N) + k_{DN}(D - N) \tag{82}$$

Assume unit amplitude: $M = e^{i\theta_M}, S = e^{i\theta_S}, N = e^{i\theta_N}, E = e^{i\theta_E}, D = e^{i\theta_D}$

Equating Real and Imaginary Parts for M

$$i\dot{\theta}_M e^{i\theta_M} = e^{i\theta_M}(\alpha_M - 1) + i\beta_M e^{i\theta_M} + k_{EM}(e^{i\theta_E} - e^{i\theta_M}) + k_{DM}(e^{i\theta_D} - e^{i\theta_M}) \tag{83}$$

Divide by $e^{i\theta_M}$:

$$i\dot{\theta}_M = (\alpha_M - 1) + i\beta_M + k_{EM}(e^{i(\theta_E - \theta_M)} - 1) + k_{DM}(e^{i(\theta_D - \theta_M)} - 1) \tag{84}$$

Separate imaginary part:

$$\dot{\theta}_M = \beta_M + k_{EM}\sin(\theta_E - \theta_M) + k_{DM}\sin(\theta_D - \theta_M) \tag{85}$$

Equating Real and Imaginary Parts for S

$$i\dot{\theta}_S e^{i\theta_S} = e^{i\theta_S}(\alpha_S - 1) + i\beta_S e^{i\theta_S} \tag{86}$$

$$+ k_{MS}(e^{i\theta_M} - e^{i\theta_S}) + k_{DS}(e^{i\theta_D} - e^{i\theta_S}) + k_{NS}(e^{i\theta_N} - e^{i\theta_S}) \tag{87}$$

Divide by $e^{i\theta_S}$:

$$i\dot{\theta}_S = (\alpha_S - 1) + i\beta_S + k_{MS}(e^{i(\theta_M - \theta_S)} - 1) + k_{DS}(e^{i(\theta_D - \theta_S)} - 1) + k_{NS}(e^{i(\theta_N - \theta_S)} - 1) \tag{88}$$

Separate imaginary part:

$$\dot{\theta}_S = \beta_S + k_{MS}\sin(\theta_M - \theta_S) + k_{DS}\sin(\theta_D - \theta_S) + k_{NS}\sin(\theta_N - \theta_S) \tag{89}$$

Equating Real and Imaginary Parts for N

$$i\dot{\theta}_N e^{i\theta_N} = e^{i\theta_N}(\alpha_N - 1) + i\beta_N e^{i\theta_N} \tag{90}$$

$$+ k_{SN}(e^{i\theta_S} - e^{i\theta_N}) + k_{DN}(e^{i\theta_D} - e^{i\theta_N}) \tag{91}$$

Divide by $e^{i\theta_N}$:

$$i\dot{\theta}_N = (\alpha_N - 1) + i\beta_N + k_{SN}(e^{i(\theta_S - \theta_N)} - 1) + k_{DN}(e^{i(\theta_D - \theta_N)} - 1) \tag{92}$$

Separate imaginary part:

$$\dot{\theta}_N = \beta_N + k_{SN}\sin(\theta_S - \theta_N) + k_{DN}\sin(\theta_D - \theta_N) \tag{93}$$

Phase Reduced Model

$$\dot{\theta}_E = \beta_E \tag{94}$$

$$\dot{\theta}_D = \beta_D \tag{95}$$

$$\dot{\theta}_M = \beta_M + k_{EM}\sin(\theta_E - \theta_M) + k_{DM}\sin(\theta_D - \theta_M) \tag{96}$$

$$\dot{\theta}_S = \beta_S + k_{MS}\sin(\theta_M - \theta_S) + k_{DS}\sin(\theta_D - \theta_S) + k_{NS}\sin(\theta_N - \theta_S) \tag{97}$$

$$\dot{\theta}_N = \beta_N + k_{SN}\sin(\theta_S - \theta_N) + k_{DN}\sin(\theta_D - \theta_N) \tag{98}$$
